# Experimental Investigation and ANFIS-Based Modelling During Machining of EN31 Alloy Steel

**DOI:** 10.3390/ma13143137

**Published:** 2020-07-14

**Authors:** Ishwer Shivakoti, Lewlyn L. R. Rodrigues, Robert Cep, Premendra Mani Pradhan, Ashis Sharma, Akash Kumar Bhoi

**Affiliations:** 1 Department of Mechanical Engineering, Sikkim Manipal Institute of Technology, Sikkim Manipal University, Majhitar 737136, India; premendramani@gmail.com (P.M.P.); ashz_1974@yahoo.com (A.S.); 2 Department of Humanities and Management, Manipal Institute of Technology, Manipal Academy of Higher Education, Mangalore 576104, India; rodrigusr@gmail.com; 3 VSB-TU Ostrava, Faculty of Mechanical Engineering, 17. listopadu 2172/15, 708 00 Ostrava, Czech Republic; robert.cep@vsb.cz; 4 Department of Electrical and Electronics Engineering, Sikkim Manipal Institute of Technology, Sikkim Manipal University, Majhitar 737136, India; akash.b@smit.smu.edu.in

**Keywords:** alloy steel, feed, ANFIS, RPM, turning

## Abstract

This research presents the parametric effect of machining control variables while turning EN31 alloy steel with a Chemical Vapor deposited (CVD) Ti(C,N) + Al_2_O_3_ + TiN coated carbide tool insert. Three machining parameters with four levels considered in this research are feed, revolutions per minute (RPM), and depth of cut (a_p_). The influences of those three factors on material removal rate (MRR), surface roughness (Ra), and cutting force (Fc) were of specific interest in this research. The results showed that turning control variables has a substantial influence on the process responses. Furthermore, the paper demonstrates an adaptive neuro fuzzy inference system (ANFIS) model to predict the process response at various parametric combinations. It was observed that the ANFIS model used for prediction was accurate in predicting the process response at varying parametric combinations. The proposed model presents correlation coefficients of 0.99, 0.98, and 0.964 for MRR, Ra, and Fc, respectively.

## 1. Introduction

Metal cutting plays a crucial role in the field of manufacturing. It was found that turning factors such as feed, depth of cut, and speed influence material removal rate (MRR) and surface roughness (RA) [1]. However, most of these research findings are established with three levels of parameters and are for specific levels of these parameters. Thus, there is adequate scope for extensive research which takes into account extended boundary conditions. The Taguchi orthogonal array has emerged as one of the most widely adopted experimental designs owing to its ability in providing the most optimal parametric combinations [2]. Generalizations of the behaviors of the factors mentioned above and the specified process performance are difficult because the findings of the earlier researchers were for specific tool–material combinations, such as the range of process variables and given turning conditions. The feed rate indicates an effect on cutting force (FC) and RA. Moreover, the influence on depth of cut was rather low while performing a turning operation on AA2219-TiB_2_/ZrB_2_ in a setup of a metal matrix composite using uncoated tungsten carbide inserts. The developed linear regression equation also showed a reliable agreement with the confirmation experiment during the turning operation [3]. The surface quality was obtained at low feed and depth of cut, while the speed was high. Increase in RA was obtained when the machining time was high during machining Inconel 718 superalloy using cemented carbide inserts [4]. Carbide tool coated with TiAlxN super nitride shows thermal stability at high temperatures while turning AISI 52,100 steel with cemented carbide inserts coated with the HSN^2^ tool. Furthermore, the depth of cut, speed, and feed have impacts on forces, RA, and wear of the insert [5]. Using minimum quantity lubrication (MQL) during machining increases the tool life and improves the quality of the surface generated when related to dry machining. Furthermore, high cutting speed can be utilized when machining with MQL [6]. The speed was the most substantial factor followed by depth of cut and feed rate, but nose radius does not show a significant effect towards RA during machining of hybrid metal matrix (Al-SiCp-fly ash) composite with uncoated tungsten carbide inserts. The optimal parametric combination obtained from the genetic algorithm shows better outcomes as compared to experimental results [7]. Grey relational analysis can be used to develop and to identify the RA of work material temperature of surface and vibration of the tool. A lower grey relational grade (GRG) value provides a smooth surface, whereas a higher GRG value increases the RA of the material [8]. Cryogenic machining provides environmentally clean machining and is successfully used in different machining processes which improve the machining performance. The turning operation in the cryogenic environment increases the MRR and flank wear through intensification in cutting velocity, feed rate, and depth of cut [9]. The introduction of a magnetic field to turning process responses, namely, radial force, FC, and feed force, results in higher results when compared to a turning operation without a magnetic field. Additionally, the increase in tool life was observed when machining was performed with a magnetic field [10]. The ANOVA-TOPSIS approach can be suitably used for determining the optimal parametric combination. It was found that the higher the MRR with a good surface, the lesser the machining temperature and the FC. The most relevant factor having the highest influence towards machining response criteria is the depth of cut [11]. When machining AISI 304 steel with uncoated carbide inserts, the RA is dominated by feed rate. The optimization of turning parameters shows a reduction in machining cost and design process [12]. The fuzzy model with adaptive networks is called ANFIS, which provides some merits over neural networks [13]. The wear in abrasive of a coated sample can sufficiently and accurately be predicted with the ANN ANFIS [14]. The objective of this research is to present the experimental investigation and ANFIS-based modelling during a turning operation of EN31 alloy steel. It was observed that the proposed ANFIS model used for prediction was accurate in predicting the process response at different parametric combinations. Optimization of turning parameters during a hard turning process using teaching–learning and bacterial foraging-based optimization shows an efficient method for determining the optimal turning parameter settings. The teaching–learning algorithm-based optimization is preferred due to its convergence at the shortest possible time [15]. The hybrid approach of ANFIS vibration and communication particle swarm optimization (VCPSO) for tool wear estimation and obtaining optimal cutting parameter settings shows that the proposed model can estimate the tool wear in real-time, which improves the efficiency of the machining process and raises the tool life [16]. The proposed research work may be extensively used in the field of metal cutting and the manufacturing environment. Subtractive clustering with ANFIS model can be used for predicting the 2nd law efficiency and total irreversibility with high accuracy as compared to ANN [17]. The hybrid approach of PSO-ANFIS and MNR algorithm is adept for estimating the CNs of diesel, and biodiesel oils have a novel correlation with high accuracy [18]. The ANFIS model utilized for automation of friction stir welding, further GA and PSO has been implemented to tune the parameters of ANFIS for better prediction. The developed ANFIS-GA and ANFIS-PSO have been observed to closely accord with the experimental results [19]. The ANFIS model can foresee more precisely when compared to ANN and other semi-empirical models using AAEDM of D3 steel [20]. The ANFIS model was tested during the investigation of FDM parameters regarding the mechanical properties of end-use parts. The developed model depicts an error percentage of 2.63%, which validates the experimental data [21]. ANFIS can be implemented as a booming technique for predicting the performance of rubberized concrete. ANFIS with Gaussian Membership functions (MFs) can predict with better accurately. The relationships amongst the parameters and strength are commonly nonlinear and well taken by the ANFIS [22]. The comparison of RSM with ANFIS model during friction stir welding of AA2024-AA5083 aluminum alloys in relation to ultimate tensile strength shows that the developed ANFIS model is a powerful method as compared to the RSM model [23]. ANFIS with fuzzy inference systems (FIS), such as subtractive clustering, grid partition, and fuzzy c-means (FCM), was utilized for determining the cetane number. The result shows that all the fuzzy inference systems can determine the cetane number of fuel (FCM), and the grid partition shows higher desirability [24]. The adsorption process modelled with ANFIS shows a minimal error of total average error and total average of absolute error, and the coefficients of determination of the training data set were found to be 0.9999 or 0.9823 respectively, when estimating the efficacy of lead adsorption with functional nanocomposite adsorbent of hydroxyapatite (HAp)/chitosan [25]. The FFA and GA utilized for optimizing ANFIS parameters during bench blasting show that both GA and FFA are capable optimizers for improving the ANFIS prediction [26]. The ANFIS model implemented for prediction of vapor compression refrigeration system shows good agreement with experimental data and shows better statistical prediction efficiency [27]. The introduction of FEA optimizer with ANFIS parades a parsimonious modelling for streamflow forecasting by integrating a small number of factors essential to return the relatively strengthen performance [28]. The ANFIS model developed for estimating carbon dioxide loading abilities of amino acid salt solutions depicts that the developed model is sufficient to estimate the loading capabilities of CO_2_ of amino acid salt solution [29]. The shear impact of the FRP modeled with the help of ANFIS shows better performance when related with seven widely used prediction tips. Further, the ANFIS model shows effective correlation with the experimental data [30]. The optimal combination obtained from utility function minimization multi criteria optimization approach shows higher material removal rate and lower feed and normal force. Further, the reduction in tool deflection and cutting time, and improvement in surface finish and tool life, were observed [31]. The cryogenic liquid nitrogen was found to be more efficient for specific energy and temperature reduction and improvement in surface quality during machining of Ti-6Al-4V. The Grey–Taguchi hybrid approach has been utilized for obtaining the optimal parametric combination [32].

## 2. Materials and Methods

The experimentation was performed on Panther Geared Centre Lathe (Gujurat Lathe MFG.Co.Pvt.Ltd, Shapar, Gujarat, India). Cutting variables which include revolutions per minute (RPM), feed, and depth of cut (a_p_) having four levels, were adopted for experimentation. The selection of the input parameters and their levels was done considering the literature survey and trial experiments. The diameter of the work material considered was 150 mm with a length of 1500 mm. The rpm values were selected based on the pilot experiment. More than 20 pilot experiments were conducted to choose the levels of each parameter. Table 1 depicts the parameters with their levels.

The Taguchi L16 array design of experimental design was implemented for designing the experiments. The process response parameters, which include MRR, Ra, and Fc, were estimated at distinct parametric settings. The turning operation was performed in EN31 alloy steel using CVD Ti(C,N) + Al_2_O_3_ + TiN coated carbide tool insert. Table 2 presents the physical properties of EN31 alloy steel.

The MRR was evaluated with an analytical equation—MRR = πdDfN, where d remains the diameter of the work piece, a_p_ is the depth of the cut, f is the feed, and N is the RPM. Ra was measured with a roughness tester, (Surftest SJ-210; Mitutoyo, Japan) the measurement was done at three different points in the finished sample with three repetitions, and the average of the same was utilized for further analysis; and Fc were measured using the dynamometer (model No-621C, N.K. Instrument, Thane, Maharashtra, India, having range—500 kgf). Table 3 provides experimental values. The experiment was repeated several times until the values converged and average values have been considered for the analysis.

## 3. Results and Discussion

The results achieved in this research work are presented in this section. The analysis of the MRR is presented in Section 3.1. The RA experimental results are presented in Section 3.2 and the evaluation of the FC tests is presented in Section 3.3.

### 3.1. Analysis of Material Removal Rate

**Figure 1:**
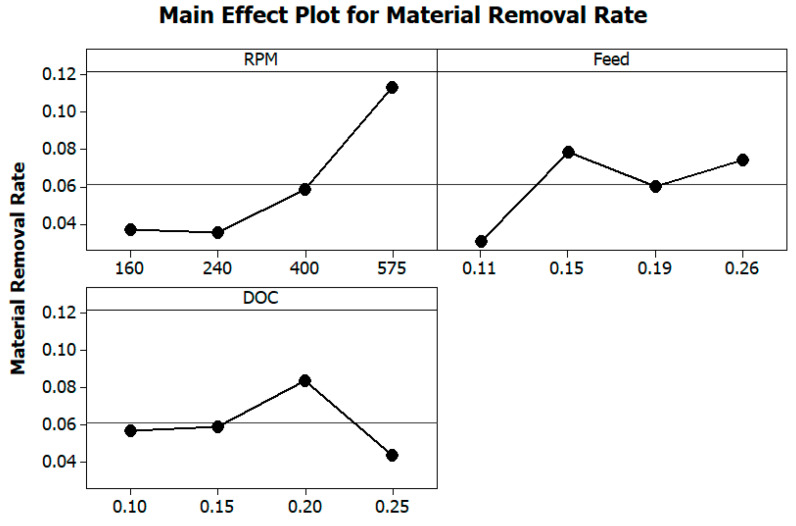
Effects of turning parameters on material removal rate (MRR).

Figure 1 presents the effect of turning parameters on MRR. The figure defines that with the rise in the rotational speed of a workpiece, the MRR increases. This is associated with an increase in RPM that leads to a rise in material removal per unit time. Moreover, with the intensification in RPM, the MRR also increases. With the increment in feed, the MRR increases in the beginning, and then, it decreases. The feed rate increases the amount of material removal per unit time. With rise in the value of feed, the MRR decreases as an increase in feed requires added cutting force, which subsequently reduces the MRR. The plot also depicts that the increment in the depth of cut initially does not have a noteworthy effect on MRR. However, the MRR increases when the depth of cut also increases. Furthermore, this is because the tool plunges into more material, which increases MRR.

**Figure 2:**
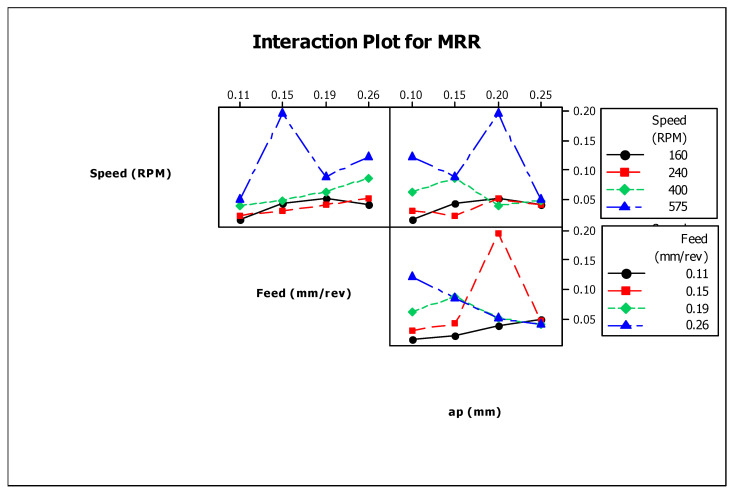
Interaction plot for material removal rate (MRR).

Figure 2 shows the interactions of MRR with other process parameters during turning. Moreover, it shows that there are influences of turning parameters on MRR at the considered values of turning parameters.

The ANOVA for the MRR is depicted in Table 4. The table presents the performance criteria with calculated F-values concerning the respective control factors.

**Figure 3:**
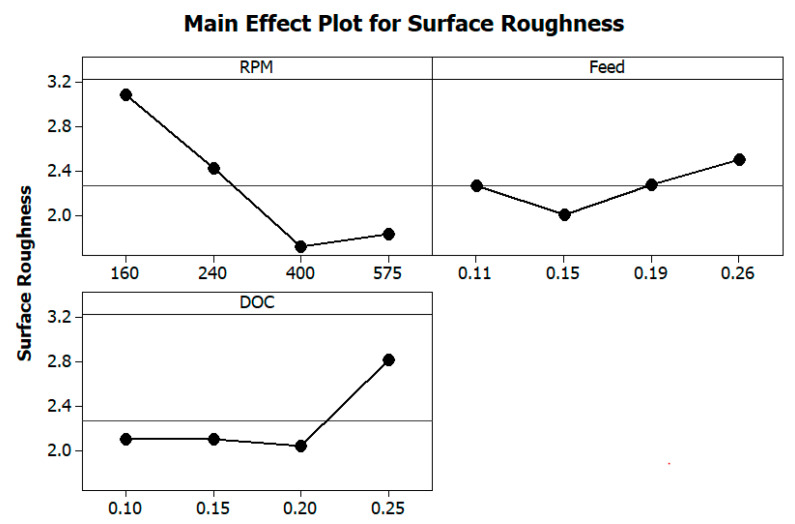
Effects of turning parameters on surface roughness (RA).

It was observed that both rpm and feed have major contributions (69% and 24% respectively) to the MRR. The contribution of error is negligibly small and hence ignored. The other parameters (feed and depth of cut) are less significant. Percentage contributions of the process parameters on MRR as a pie chart are shown in Figure 3. The percentage contributions of the process variables on MRR as a chart are shown in Figure 7.

### 3.2. Analysis of Surface Roughness

The consequence of process variables on surface roughness is represented in Figure 3. It was perceived that with increment in the RPM, the roughness of a machined surface reduced. An increase in cutting speed leads to a decrease in the built-up edges, and thus, the friction force between the cutting tool and workpiece surface to be machined is much less. This is because the resistance offered by the cutting edge to cutting gets reduced with a reduction in contact area between the tool and the work surface [33]. The graph also depicts that the surface roughness increases with increases in feed rate as well. This is because during machining, the cutting edge of the tool is not able to cut uniform material from the upper surface of a rotating job. Besides, there is a reduction in the contact time during machining that leads to the material not being appropriately removed.

**Figure 4:**
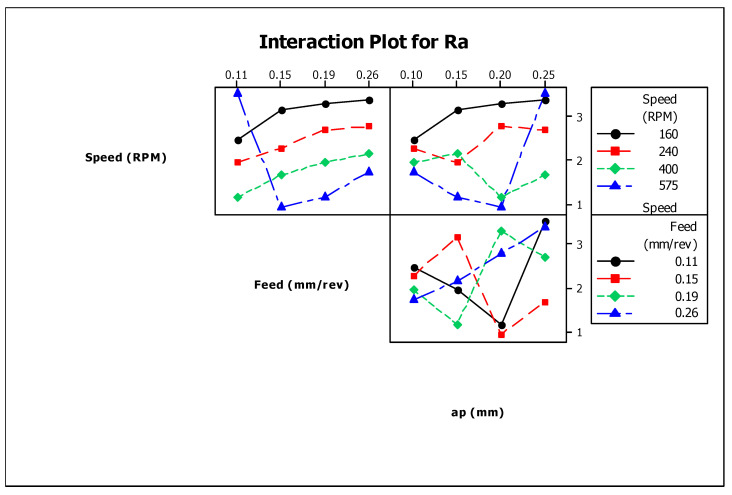
Interaction plot for surface roughness (RA).

The depth of cut does not show much significance up to a certain level. However, a greater depth of cut rises the Ra values. A higher value of depth shows a further reduction of material from the work surface, causing better surface finish as removal of material from the work surface takes place slowly. Figure 4 presents the interaction of surface roughness with other process parameters during turning operation. The figure indicates that the process parameters have huge influences on surface roughness values. Table 5 shows the ANOVA results for surface roughness and calculated F-values concerning the respective control factors. It can be observed that only cutting speed (rpm) has a statistically significant contribution (68.8%) to the surface roughness of the work-piece. The percentage contributions of the process parameters on surface roughness as a bar chart is shown in Figure 7.

### 3.3. Analysis of Cutting Force

**Figure 5:**
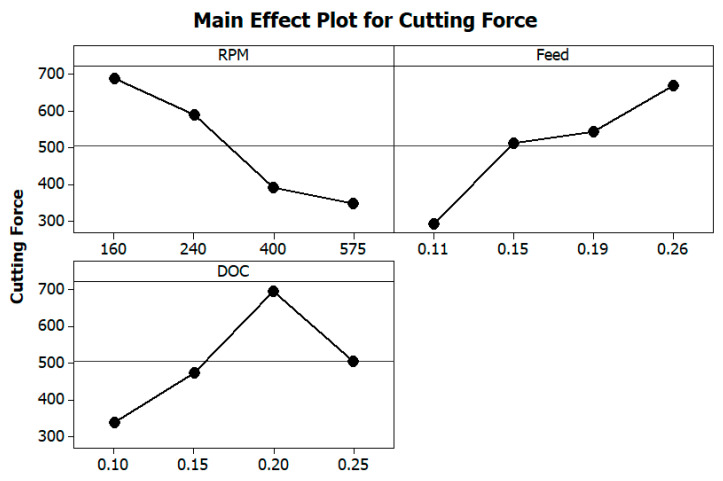
Effects of turning parameters on Cutting Force (Fc).

**Figure 6:**
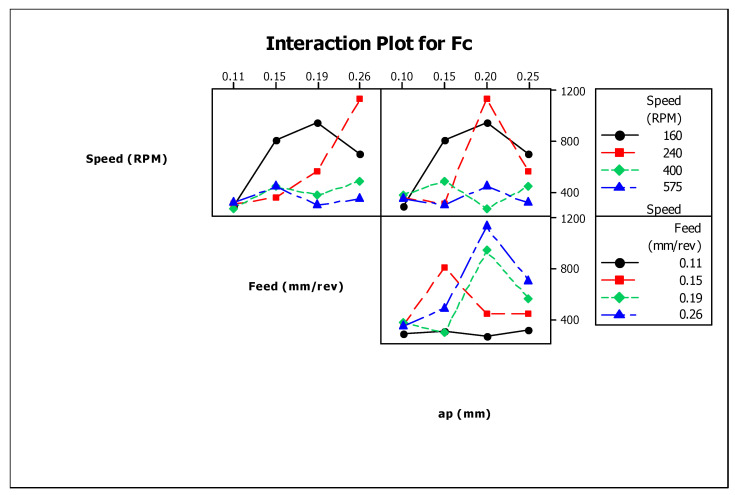
Interaction plot for cutting force (FC).

The significance of turning parameters on cutting force is represented in Figure 5. It is understood from the figure that the rise in cutting speed decreases the cutting force, which could be owing to a temperature increase at the tool–work contact. Additionally, the shear strength of the material at the cutting zone reduced at a higher value of spindle speed. The graph also shows that the increment in feed rate raises the cutting force. This is caused by the increase in chip load per tooth and the increment in feed rate [13]. The increment in the depth of cut for a specific value (0.1 to 0.2 mm) has resulted in a higher value of cutting force, and further, an additional increment in the depth of the cut decreases the force of cutting. Figure 6 depicts the interaction plot for cutting force.

**Figure 7:**
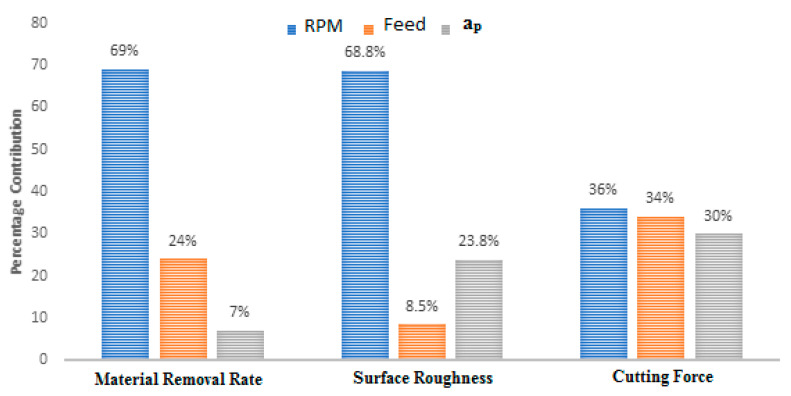
Percentage contributions of turning parameters on process response.

The ANOVA test for cutting force is presented in Table 6. Relating the F calculated and F standard, it is confirmed that rotating speed and feed process parameters are significant to the 90% confidence level. However, the depth of cut parameter is substantial at the 75% confidence level. The percentage contributions of the process parameters on cutting force are shown in Figure 7.

## 4. Adaptive Neuro Fuzzy Inference System (ANFIS)-Based Prediction Model

ANFIS is a well-known hybrid model which was discovered by Jang in 1993 [13]. Indeed, it can set up a feasible rule, which coordinates with a robust neural network system to predict the output data, as it inherits those unique and efficient features from fuzzy logic and artificial neural networks. ANFIS is a five-layered system [14], which is based on the Takagi–Sugeno fuzzy interference system [1]. Every layer possesses a different function as mentioned below:

Layer I: The most relevant layer of all the layers which converts the input set to fuzzy set takes place. The membership function plays a prime role to undergo a conversion of the input set to a fuzzy set. There are a set of adaptive nodes in a fuzzy layer whose functions are described as:

A1,x = µBx (P) (x = 1,2)(1)

A1,y = µCy (Q) (y = 1,2)(2)

A1,z = µDz (R) (z = 1,2)(3)

P, Q, and R are input variable nodes. x, y, z, B, C, and D are labels connected with variable input nodes having µ(P), µ(Q) and µ(R) as membership functions. Due to the versatility, Gaussian shape is selected in this case out of all membership function.

Layer II: The output layer is a fixed function of input signal and the node function and is represented as:

A2,x = Gx = µBx (P). µCy (Q). µDz (R), (for x,y and z = 1,2)(4)

A2 represents the output of layer II, and Gx corresponds to the fuzzy strength rule.

Layer III: It has a defined function node network like layer II and layer strength III depicted as:

A3,x = Gl = Gx/(G1 + G2), (for x = 1,2)(5)

A3 represents the output of layer III, and Gl is the normalized strength rule for layer III.

Layer IV: The nature of this is layer is inconsistent and varies spontaneously. Due to the nature of the layer node being adaptable, and the strength function is defined as:

A4,x = Glx.fx, (for x = 1,2)(6)

Here, Glx is the normalized weight of xth rule. fx is a fuzzy rule for x = 1,2. Therefore the fuzzy rule setup is evaluated as:

Fx = (Uxl1 + Vxl2 + Wx)(7)

Ux, Vx, and Wx are the defined parameters.

Layer V: Total outcome is calculated in this layer. It has a fixed node, and the total output of the total system is evaluated by:

A5,x = ΣxGnx.fx(8)

The training of input values was done by applying the backpropagation method and was tested based upon the trained values.

The Gaussian membership function was selected and applied due to its versatility and reliability. The root mean square error (RMSE), correlation coefficient (R), and mean absolute percentage error (MAPE) of the predicted values were evaluated by using the following relations [1]:

(9) $$\mathrm{MAPE}=\frac{100\%}{T_{no}}\sum_{t=1}^{T_{no}}\left|\frac{E_{V}-P_{V}}{E_{V}}\right|$$

(10) $$\mathrm{RMSE}=\sqrt{\frac{1}{T_{no}}\times \sum_{j=1}^{T_{no}}{\left(E_{V}-P_{V}\right)}^{2}}$$

(11) $$R=\sum \frac{\left(\left(E_{V}-M_{PV}\right)\left(P_{V}-M_{EV}\right)\right)}{\sqrt{\left(\sum {\left(E_{V}-M_{EV}\right)}^{2}\left(\sum {\left(P_{V}-M_{PV}\right)}^{2}\right)\right)}}$$

Here *E_V_* is the experimental value, *P_V_* is the predicted value, *M_EV_* is the mean of the experimental value, *M_PV_* is the mean of the predicted value, and *T_no_* is the number of experiments.

The experimental results and predicted values of MRR, Ra, and Fc are represented in Table 7.

**Figure 8:**
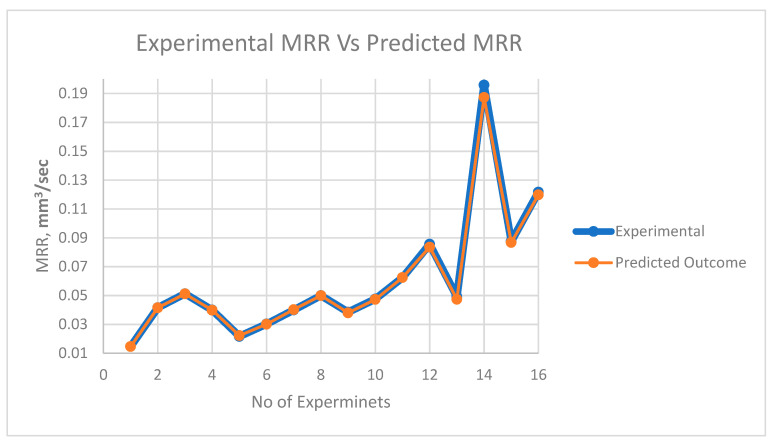
Plot of experimental MRR vs. predicted MRR.

**Figure 9:**
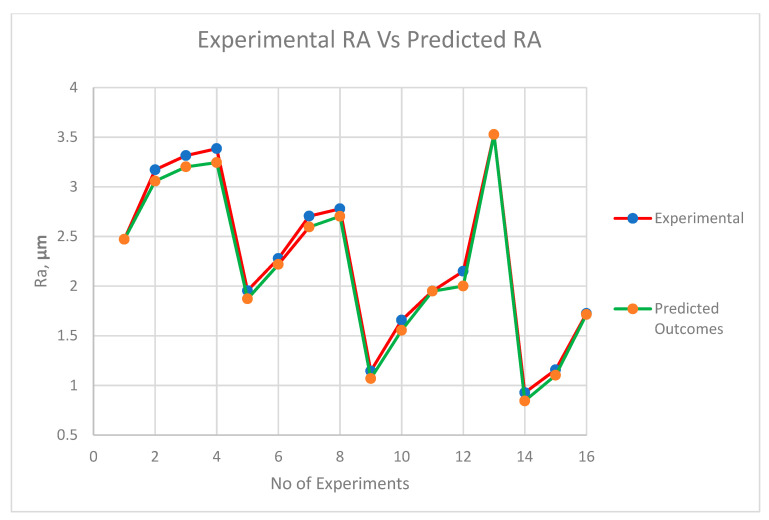
Plot of experimental surface roughness (Ra) vs. predicted surface roughness (Ra).

**Figure 10:**
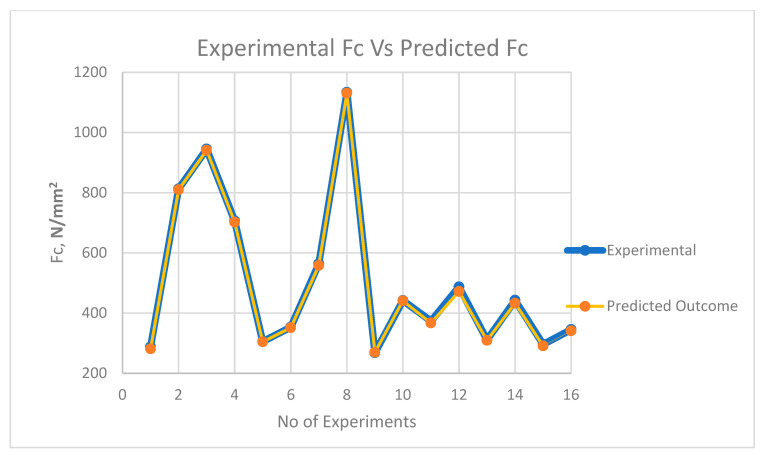
Plot of experimental cutting force (Fc) vs. predicted cutting force (Fc).

The comparative plot of predicted values based upon the ANFIS model and experimental values is shown in Figure 8, Figure 9 and Figure 10. It is witnessed from the plot that ANFIS-based predicted data show reliable responses concerning experimental values for all three outputs. Hence, the proposed ANFIS model is accurate for predicting the output values for varying parametric combinations.

Furthermore, Table 8 depicts the values of MAPE, RMSE, and R for all the experimental and predicted values.

The parametric analysis was performed considering three process factors, namely, RPM, feed, and depth of cut, and three process performance variables—MRR, Ra, and Fc. It was observed that the cutting parameters influence the process characteristics. The RPM is the most promising factor for all the performance parameters, namely, MRR, Ra, and Fc. The correlation coefficient suggested that the prediction model is reliable and can be utilized for predicting the output responses, such as MRR, Ra, and Fc [1].

The ANFIS-based prediction was undertaken in this research to predict the process response values at different parametric combinations. The research concludes that the proposed ANFIS model used for prediction is accurate at predicting the process responses at varying variable combinations. The developed model is useful for predicting the values of performance criteria at different parametric combinations in a turning operation. Machining of EN31 alloy steel, which is high carbon alloy steel, has been a critical area of study in the of metal cutting domain in the past several years. Moreover, it is related to a wide range of applications, such as round bars, necessarily, the connectors between pipes in oil and gas systems, water supply systems, and plugs to resist pressure in pressure valves, which are made available at lengths of 100 mm to 6000 mm with a diameter range from 5 mm to 500 mm. Despite the wide range of applications, the machining of EN31 alloy steel has always been a challenge due to its physical properties, especially the hardness.

## 5. Conclusions

The research presents an experimental investigation and ANFIS-based modelling during the turning operation of EN31 alloy steel. The major findings of the research work are as follows.

1. The proposed model presents R values of 0.99, 0.98, and 0.964 for MRR, Ra, and Fc, respectively.
2. The RPM is the major contributing parameter for all performance responses, such as MRR, Ra, and Fc.
3. The proposed ANFIS model is suitable for predicting the performance responses at all values of parametric combinations.
4. Machining of EN 31 alloy steel, which is a high carbon alloy steel, has been an essential area of research in the field of metal cutting in the past several years owning to its wide range of applications, such as: round bars essentially as the connectors between pipes in oil and gas systems, water supply systems, and plugs to resist pressure in pressure valves. Moreover, this material is available at lengths from 100 mm to 6000 mm with a diameter range from 5 to 500 mm. Despite the wide range of applications, the machining of EN 31 alloy steel has always been a challenge due to its physical properties, especially the hardness. Therefore, machining parameter optimization becomes a critical area of research and this research has contributed to the body of knowledge in this area. The findings of this research may be useful for the manufacturers and future researchers in this area.

## Figures and Tables

**Table 1:** Turning parameters and their levels.

Sl. No.	RPM	Feed (mm/rev)	a_p_ (mm)
1	160	0.11	0.1
2	240	0.15	0.15
3	400	0.19	0.2
4	575	0.26	0.25

**Table 2:** EN31 alloy steel physical properties.

Density	7850 $\mathrm{kg}/m^{3}$
Thermal Conductivity	46.6 W/mK
Tensile Strength	750 $N/{\mathrm{mm}}^{2}$
Yield Stress	450 $N/{\mathrm{mm}}^{2}$
Modulus of Elasticity	215000 $N/{\mathrm{mm}}^{2}$
Hardness	63 HRc

**Table 3:** Experimental results.

Sl. No	RPM	Feed (mm/rev)	a_p_ (mm)	MRR (mm^3^/sec)	Ra (µm)	Fc (N/mm^2^)
1	160	0.11	0.1	0.0147	2.472	288.04
2	160	0.15	0.15	0.0416	3.172	812.63
3	160	0.19	0.2	0.0513	3.315	944.84
4	160	0.26	0.25	0.04	3.385	706.32
5	240	0.11	0.15	0.0217	1.954	305.58
6	240	0.15	0.1	0.0303	2.278	353.28
7	240	0.19	0.25	0.04	2.705	564.00
8	240	0.26	0.2	0.05	2.779	1133.34
9	400	0.11	0.2	0.0385	1.145	267.93
10	400	0.15	0.25	0.0476	1.657	441.84
11	400	0.19	0.1	0.0625	1.950	373.61
12	400	0.26	0.15	0.0856	2.150	487.07
13	575	0.11	0.25	0.0492	3.528	311.26
14	575	0.15	0.2	0.1958	0.927	442.69
15	575	0.19	0.15	0.0875	1.158	296.10
16	575	0.26	0.1	0.1217	1.724	345.38

**Table 4:** Analysis of variance for material removal rate (MRR).

Source	Degree of Freedom	Sequential Sums of Squares	Adjusted Sums of Squares	Adjusted Mean Squares	F Value	% Contribution
RPM	3	0.0159940	0.0159940	0.0053313	6.12	69
Feed	3	0.0055767	0.0055767	0.0018589	2.13	24
a_p_	3	0.0032955	0.0032955	0.0010985	1.26	7
Error	6	0.0052278	0.0052278	0.0008713	-	-
Total	15	0.0300941	-	-	-	-

Standard value of F distribution: F_0.25_, 3,6 = 1.78; F_0.1_, 3,6 = 3.288; F_0.05_, 3,6 = 4.757; F_0.025_, 3,6 = 6.599; F_0.01_, 3,6 = 9.780.

**Table 5:** Analysis of Variance for Surface Roughness (RA).

Source	Degree of Freedom	Sequential Sums of Squares	Adjusted Sums of Squares	Adjusted Mean Squares	F Value	% Contribution
RPM	3	4.7100	4.7100	1.5700	2.63	68.8
Feed	3	0.5036	0.5036	0.1679	0.28	8.5
a_p_	3	1.6252	1.6252	0.5417	0.91	23.8
Error	6	3.5781	3.5781	0.5964	-	-
Total	15	10.4169	-	-	-	-

Standard value of F distribution: F0.25, 3,6 = 1.78; F0.1, 3,6 = 3.288; F0.05, 3,6 = 4.757; F0.025, 3,6 = 6.599; F0.01, 3,6 = 9.780.

**Table 6:** Analysis of variance for cutting force (FC).

Source	Degree of Freedom	Sequential Sums of Squares	Adjusted Sums of Squares	Adjusted Mean Squares	F Value	% Contribution
RPM	3	310203	310203	103401	3.82	36
Feed	3	292275	292275	97425	3.60	34
a_p_	3	260099	260099	86700	3.20	30
Error	6	162572	162572	27095	-	-
Total	15	1025148	-	-	-	-

Standard value of F distribution: F_0.25_, 3,6 = 1.78; F_0.1_, 3,6 = 3.288; F_0.05_, 3,6 = 4.757; F_0.025_, 3,6 = 6.599; F_0.01_, 3,6 = 9.780.

**Table 7:** Experimental and predicted results.

SR	Experimental Outcome			Predicted Outcome		
	MRR	Ra	Fc	MRR	Ra	Fc
1	0.0147	2.472	288.04	0.0147	2.471	281.20
2	0.0416	3.172	812.63	0.0416	3.058	810.00
3	0.0513	3.315	944.84	0.0512	3.202	941.00
4	0.04	3.385	706.32	0.0399	3.245	702.45
5	0.0217	1.954	305.58	0.0223	1.872	304.65
6	0.0303	2.278	353.28	0.0302	2.219	351.45
7	0.04	2.705	564	0.0403	2.595	558.00
8	0.05	2.779	1133.34	0.0500	2.704	1130.57
9	0.0385	1.145	267.93	0.0380	1.070	269.56
10	0.0476	1.657	441.84	0.0473	1.555	440.74
11	0.0625	1.95	373.61	0.0625	1.949	367.14
12	0.0856	2.15	487.07	0.0835	2.001	472.06
13	0.0492	3.528	311.26	0.0474	3.527	309.45
14	0.1958	0.927	442.69	0.1873	0.844	432.91
15	0.0875	1.158	296.1	0.0868	1.102	290.92
16	0.1217	1.724	345.38	0.1198	1.715	341.06

**Table 8:** Absolute percentage error (MAPE), root mean squared error (RMSE), and correlation coefficient (R).

Error	MRR	Ra	Fc
MAPE	1.18	3.67	3.62
RMSE	0.002	0.087	5.845
R	0.99	0.98	0.964
